# A Pilot Study to Describe Cardiometabolic Health Indicators and Prescription Medication Use in Postmenopausal Women with a Self-Reported History of PMOS †

**DOI:** 10.3390/jcm15145533

**Published:** 2026-07-15

**Authors:** Emily J. Arentson-Lantz, Mariel K. Miller, Crystal C. Douglas

**Affiliations:** 1 Department of Nutrition Sciences and Health Behavior, University of Texas Medical Branch, Galveston, TX 77555-1124, USA; ejlantz@utmb.edu; 2 Center for Health Promotion, Performance, and Rehabilitation Research, University of Texas Medical Branch, Galveston, TX 77555-1124, USA; 3 Institute of Translational Science, University of Texas Medical Branch, Galveston, TX 77555-1124, USA; makmille@utmb.edu

**Keywords:** polypharmacy, PCOS, polycystic ovary syndrome, polyendocrine metabolic ovarian syndrome, PMOS, cardiometabolic, adiposity, menopause, medication, quality of life

## Abstract

**Background/Objectives:** Relatively little is known about how the aberrations in cardiometabolic health linked with polyendocrine metabolic ovarian syndrome (PMOS, formally called polycystic ovary syndrome or PCOS) persist beyond the menopause transition. The study aim was to explore and contrast cardiometabolic health indicators in postmenopausal women with PMOS with postmenopausal women without PMOS. This study employed a cross-sectional analysis; comparisons between groups were completed with a two-tailed *t*-test. **Methods:** Postmenopausal females with a self-reported history of PMOS/PCOS (n = 10; 58.0 ± 5.1y; P) and without a self-reported history of PMOS/PCOS diagnosis (56.5 ± 3.6 n = 19; y; CON) completed a study visit to assess indicators of cardiometabolic health, including anthropometric measurements, body composition, sex hormones, fasting blood lipids, glucose, insulin, and glycosylated hemoglobin. Medical diagnosis of PMOS, menstrual health history, medication usage, and quality-of-life indicators were self-reported. **Results:** Postmenopausal women with PMOS had a significantly higher BMI (CON vs. PMOS: 25.8 ± 5.3 kg/m^2^ vs. 29.0 ± 3.1 kg/m^2^, *p* < 0.045), ratio of trunk fat to appendicular fat mass (CON vs. PMOS, 1.20 ± 0.39 vs. 1.40 ± 0.21; *p* = 0.041), and tended to have a higher waist-to-hip ratio (*p* = 0.089). Circulating sex hormones, glucose, and insulin were not significantly different between the two groups (*p* > 0.05). However, women with PMOS tended to report a higher number of prescription medications (n = 9; *p* = 0.058), with all PMOS participants reporting at least one prescription medication, while 36.8% of CON participants reported no prescription medications. **Conclusions:** These preliminary findings suggest that postmenopausal women with a self-reported history of PMOS may have greater central fat distribution and higher prescription medication use than controls. Larger, more diverse studies with confirmed PMOS diagnosis and detailed medication indication data are needed before clinical conclusions can be drawn.

## 1. Introduction

Polyendocrine metabolic ovarian syndrome (PMOS), formally called polycystic ovary syndrome (PCOS), is the most common endocrine disease diagnosed in women of reproductive age. While rates of PMOS in the United States vary with diagnostic criteria, between 5 and 20% of women are affected [1]. The Rotterdam ESHRE/ARM Consensus Workshop held in 2003 established the most widely implemented PMOS diagnostic criteria that require the presence of two or more of the following indications to establish a diagnosis: polycystic ovarian morphology, presence of biochemical or clinical hyperandrogenism (HA), or oligo-anovulation [2,3]. Thus, PMOS includes four unique phenotypes, emphasizing the heterogeneity of the condition. Clinical features of PMOS related to HA frequently include hirsutism, acne, or menstrual irregularity, contributing to increased risk for anxiety and depression, and reduced quality of life (QoL) [4]. Additionally, metabolic features of PMOS, such as obesity, particularly visceral obesity, dyslipidemia, and insulin resistance (independent of obesity/body weight), increase risks for the development of chronic diseases [5,6,7]. A recent systematic review and meta-analysis of 23 studies with 945 women with PMOS across the lifespan noted that women with PMOS had a higher risk of type 2 diabetes and hypertension compared to women without PMOS, as well as dyslipidemia and an increase in non-fatal cerebrovascular disease events [8]. Notably, the authors note the large heterogeneity in PMOS diagnostic criteria employed and that findings were primarily from Caucasian women, potentially limiting generalizability.

Despite the well-documented severe, chronic impact that PMOS has on women’s physical and emotional health during the reproductive years, relatively little is known about how the syndrome evolves as women age [9]. As women with PMOS approach menopause, the syndrome is largely thought to transition from a reproductive disorder to a metabolic disorder [10]. The onset of menopause and the associated drop in circulating levels of metabolically protective estrogen are characterized by a decline in cardiometabolic health [11,12,13,14], but the additional metabolic burden of PMOS during the menopause transition is not yet well described. There are relatively few well-controlled longitudinal studies that track health outcomes in aging women with PMOS [15]. Some evidence suggests an increase in the prevalence of visceral obesity, dyslipidemia, type 2 diabetes, hypertension, and cardiovascular risk factors in older women with PMOS when compared to age-matched women without a history of PMOS [16,17,18]. Consequently, international guidelines for clinical management of PMOS recommend continued screening for cardiometabolic risk factors beyond the reproductive years [19].

Building a more robust understanding of the cardiometabolic health of women with PMOS following the menopause transition is critical to better inform their care. Therefore, the objective of this pilot study was to explore and contrast cardiometabolic health indicators, self-reported medication usage, and quality-of-life indicators in postmenopausal women with and without polyendocrine metabolic ovarian.

## 2. Methods

We conducted an exploratory pilot study to collect anthropometric, body composition, blood lipids, sex hormones, glucose, insulin, and self-reported medication usage in postmenopausal women with and without a self-reported history of polyendocrine metabolic ovarian syndrome. Data were collected between March 2023 and August 2024. The pilot study sample size was restricted due to resource availability, but was considered adequate to evaluate feasibility, including recruitment of the postmenopausal group with PMOS.

### 2.1. Participants

Postmenopausal women with a self-reported history of PMOS/PCOS (PMOS; n = 10) or without a self-reported history of PMOS/PCOS (CON; n = 19) were recruited from the greater Galveston/Houston, TX area utilizing digital advertising, flyers, and provider referrals. All participants completed a telephone screening to confirm eligibility. Eligible participants were ≥50 years, self-reported postmenopausal status according to STRAW criteria [20] (no menstruation within the previous 12 months or longer), and provided height and weight data consistent with a body mass index (BMI) between 18.5 and 40 kg/m^2^. Exclusion criteria included current smokers, alcohol abuse, recent (within 3 years) history of cancer treatment (excluding basal carcinoma), surgical or pharmacologically induced menopause (e.g., hysterectomy or oophorectomy or chemically induced menopause), and recent (within 3 months) use of hormone therapy. All participants who reported having PMOS stated they had previously received a medical diagnosis of PMOS by a healthcare provider prior to menopause; however, we were unable to confirm the PMOS diagnosis with medical records. To provide some verification for the presence of PMOS symptoms during reproductive years, a self-administered screener for PMOS was administered electronically via REDCap; participants in the control group had to score a 2 or below, indicating no presence of hirsutism [21,22]. Control participants were also evaluated for metabolic indicators of PMOS, including a history of irregular menses and acne. If participants were eligible and indicated interest in participating, the details of the study were presented during the phone call. Written informed consent was captured electronically using the e-consent module available through REDCap. All participants received monetary compensation for their time and effort. The study protocol was conducted in accordance with the Declaration of Helsinki and approved by The University of Texas Medical Branch’s (UTMB) Institutional Review Board (IRB#23-0011).

### 2.2. Study Visit and Procedures

All participants visited the UTMB Institute for Translational Science Clinical Research Center following an overnight fast. Height and weight were measured by research staff using standard procedures and equipment. Seated blood pressure was measured using an oscillometric blood pressure monitor. Waist circumference was measured with a non-elastic, flexible measuring tape just above the iliac crest at the level of the umbilicus. Hip circumference was measured with the same non-elastic flexible measuring tape at the maximum circumference of the buttocks.

Fasting blood samples were obtained with venipuncture using aseptic techniques by trained study staff and analyzed for glucose, insulin, sex hormones, glycosylated hemoglobin (HbA1C), blood lipids, and inflammatory markers by the UTMB Central Laboratory. Immunoassay techniques were used to measure total testosterone (T) and sex hormone-binding globulin (SHBG). Free androgen index (FAI), or a measure of free T, was calculated using the following equation: 100 × Total T (nmol/L)/SHBG (nmol/L) [23].

Body composition was measured with dual-energy x-ray absorptiometry (DEXA; Lunar iDXA; GE Medical Systems, Madison, WI, USA). Participants were asked to lie supine for five minutes prior to the scan.

### 2.3. Medical History and Presentation of Hirsutism

Participants were asked to briefly report on some aspects of their reproductive health history, including recalling their age of first menstruation, age of last menstruation, and age of PMOS diagnosis. They were also asked to report their current use of prescription and over-the-counter medication, including information on dosage, route of administration, and frequency of administration. Self-reported prescription drugs meeting FDA approval were coded according to the US Pharmacopeia Drug Classification (USPDC) 2025 broad categorical system, containing over 50 USP categories, not the USP class or pharmacotherapeutic group, as the therapeutic intention of the drug was not requested from participants [24]. For example, the classification of Liraglutide medication usage was coded under the USP category Blood Glucose Regulators, rather than Antidiabetic Agents or GLP-1 Receptor Agonists. Medications intended for various uses were coded under all applicable categories, unless, in the rare event, the participant provided the intended use of the prescribed drug. For example, one participant reported the use of Esomeprazole 40 mg daily post-gastric sleeve, so this drug was coded as category Gastrointestinal Agent vs. Analgesics. Estrogens were coded as Hormonal Agents (Sex Hormones), not Contraceptives, for this postmenopausal population, and medications halted for study purposes were still coded (i.e., Estrogen). Reports of multiple medications within the same USP category were counted, and the total FDA-approved drug count was calculated for all participants.

Presence or severity of hirsutism was self-assessed based on presentation during reproductive years using a simplified Ferriman–Gallwey (sFG) scale, comprising six androgen-sensitive body sites. Scores of 0–4 at each site were totaled, with higher scores representing increased severity of hirsutism [25,26]. Participants with calculated total scores of ≥ 8 were deemed hirsute [27].

### 2.4. Quality-of-Life Indicators

Participants completed several electronically administered questionnaires regarding medical history and quality-of-life indicators. Depression and anxiety symptoms were assessed with the NIH PROMIS Depression v8a and PROMIS Anxiety v8a [28,29]. Participants reported outcomes related to social function and their perceived ability and satisfaction to perform usual social roles and activities using the NIH PROMIS Ability to Participate in Social Roles v2.0 4a and Activities and PROMIS Satisfaction with Social Roles and Activities v2.0 4a [30]. We also asked questions about sleep health using PROMIS Sleep-related impairment, Sleep Disturbance, and Fatigue since the menopausal transition is associated with disrupted sleep. For the PROMIS outcomes reported here, a T score of 50 is average for the US population; a score of 10 less than or higher than 50 (e.g., 40 or 60) is considered to be lower or higher than average.

### 2.5. Statistical Analysis

Group differences in descriptive measures (anthropometrics, cardiometabolic health indicators, body composition) were determined via a two-tailed *t*-test with statistical significance set at *p* < 0.05 (unadjusted). Cohen’s d and 95% confidence interval are also reported. Medication data were not normally distributed, so a Mann–Whitney *U* test was used, and data are reported as the group median. Statistical analysis was performed using GraphPad Prism v10 (GraphPad, Boston, MA, USA).

## 3. Results

A total of 19 postmenopausal women without a self-reported history of PMOS (CON) and 10 postmenopausal women with a self-reported history of PMOS (PMOS) completed a metabolic visit with anthropometric measurements, blood draw, and body composition assessment. Participant demographics are presented in Table 1. The imbalance between the CON and PMOS groups was due to the difficulty in recruiting postmenopausal participants with a PMOS diagnosis. The self-reported age of menopause and mean years post-menopause were similar between groups (CON vs. PMOS, *p* = 0.60 and *p* = 0.22, respectively); however, the severity of hirsutism during reproductive years, evaluated using the sFG tool, was increased in the PMOS group (CON vs. PMOS, *p* = 0.0017).

### 3.1. Cardiometabolic Health Indicators

There were no statistically significant differences in height, weight, waist circumference, or hip circumference between the CON and PMOS groups (Table 2). The PMOS group did have a significantly higher BMI (*p* = 0.045), although the average BMI of both groups was classified as overweight. The waist-to-hip ratio of the PMOS group tended (*p* = 0.089) to be higher. Also of note, the mean waist circumference of both groups exceeded the recommendations linked to lower cardiometabolic risk for women (<35 in).

There were no statistically significant differences in estradiol, total T, androstenedione, SHBG, or FAI (Table 2). Total cholesterol was significantly lower in women with PMOS, but there were no significant differences in any other blood lipids, systolic or diastolic blood pressure, glucose, insulin, HbA1c, or hsCRP (Table 2).

No significant difference in total fat mass, lean mass, percent fat mass, trunk fat, and visceral fat mass measured by DEXA was observed between women with and without PMOS (Table 2). However, the ratio of trunk fat to leg fat mass and trunk fat to appendicular fat mass was significantly increased in women with PMOS, indicating differences in relative fat distribution ratios (Table 2).

**Figure 1:**
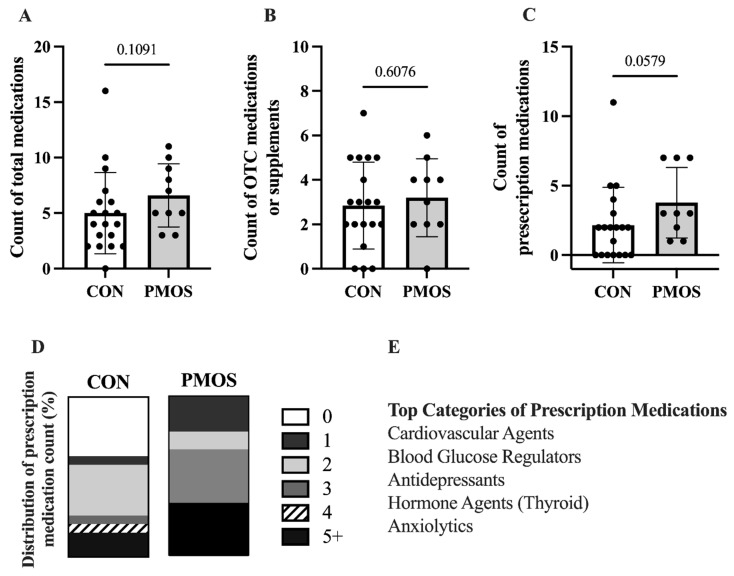
Self-reported current (within previous 6 months) prescription and over-the-counter (OTC) medication usage in postmenopausal women with (PMOS; n = 9) and without (CON; n = 19) a diagnosis of polyendocrine metabolic ovarian syndrome. Total count for combined prescription and over-the-counter medications is shown in (**A**), with OCT medications only in (**B**) and prescription medications only in (**C**). (**D**) shows the distribution of prescription medication count for both groups. (**E**) denotes the top five categories of prescription medications in descending order of the count of how many participants reported using a medication in that category. The US Pharmacopeia Drug Classification (USPDC) 2025 [24] broad categorical system was used to categorize the medications reported by participants. The data were not normally distributed, so a Mann–Whitney test was used, and medians are reported. One participant with PMOS did not complete any survey data regarding prescription medications.

The median number of total medications and over-the-counter medications was not significantly different between women with (n = 9) and without a PMOS diagnosis (Figure 1A, *p* = 0.11 and Figure 1B, *p* = 0.61, respectively). Participants with a PMOS diagnosis tended (*p* = 0.058) to report a higher median number of prescription medications (Figure 1C). While 36.8% of participants without PMOS reported no prescription medications, all participants with PMOS reported one or more prescription medications, with three out of nine participants (33%) reporting five or more prescription medications (Figure 1D). Participants in both groups most often reported a prescription medication classified as a cardiovascular agent, blood glucose regulator, antidepressant, hormone agent/regulator (thyroid), or anxiolytic (Figure 1E).

### 3.2. Quality-of-Life and Mood Indicators

There were no statistically significant differences in quality-of-life and mood indicators (PROMIS Depression, Anxiety, Ability to Participate, Social Satisfaction, Sleep Related Impairment, Sleep Disturbance, Fatigue). Data are reported in Supplementary Table S1.

## 4. Discussion

This pilot study assessed cardiometabolic health indicators in a small cohort of postmenopausal women with and without PMOS using biomarkers and body composition, self-reported medication usage, and quality-of-life indicators. Cardiometabolic health biomarkers, including most blood lipids, blood pressure, fasting glucose and insulin, and inflammatory markers, did not differ between women with and without PMOS. However, women with PMOS reported a higher number of prescription medications than those without a diagnosis of PMOS, which may indicate that postmenopausal women with PMOS rely on pharmacological treatments to manage their cardiometabolic health. Given the pilot nature of the study and the small sample size, analyses were exploratory. No correction for multiple comparisons was applied, and statistically significant findings should be interpreted cautiously. However, these findings provide valuable insight for future studies to evaluate medication management of PMOS and age-related chronic disease among older women with PMOS.

As expected, participants with PMOS reported receiving a PMOS diagnosis during the reproductive years around the third decade of life. Fernandez et al. similarly reported that the initial clinical diagnosis of PMOS received among a community-based cohort of women was made in the third decade of life [31]. Additionally, women with PMOS described a clinical presentation of hirsutism, including experiencing increased body hair. Hirsutism evaluation required participants to recall body hair presentation prior to menopause. Self-assessment of depilatory techniques or hirsutism can predict PMOS [22], though women may rate their hirsutism higher than clinicians [32]. As exogenous estrogen was an exclusion criterion for study entry, we expected to see evidence of relative HA in the PMOS group [33], though we did not assess for current depilatory practices or present-day hirsutism. While women with PMOS may experience a delay in the onset of menopause up to four years [34], the self-reported age at menopause was similar between the women with and without PMOS in this present work, likely due to the small number of participants.

The menopause transition is associated with an increase in body weight and deposition of visceral adiposity. Likewise, increased body weight is frequently associated with PMOS and appears to worsen the metabolic and hormonal features of PMOS. While we did not detect group differences in body weight, the PMOS group had a significantly higher BMI, as all participants with PMOS had a BMI classified as overweight (>25 kg/m^2^) or obese (>30 kg/m^2^). Waist circumference was greater than the current recommendations for females (>35 inches) in both groups, indicating increased central adiposity, which is associated with increased cardiometabolic health risks [35,36]. Because of emerging evidence that anthropometric measures typically measured in the clinic, including BMI and waist circumference, may not be a sufficient predictor of cardiometabolic health in older adults [37,38,39,40], we also measured body fat distribution with DEXA. Women with PMOS demonstrated greater central distribution of fat as indicated by a greater ratio of trunk-to-leg fat. Increased central adiposity relative to appendicular fat mass is associated with increased risk for dyslipidemia [41], high blood pressure, diabetes [42], metabolic syndrome and mortality among older adults, independent of BMI and waist circumference [42]. Our data suggest that postmenopausal women with PMOS may exhibit body adiposity measures that indicate they are at increased risk for cardiometabolic diseases, which may not be detected by traditional anthropometric measurements. Therefore, a more comprehensive evaluation of cardiometabolic health merits consideration; this includes further testing for indicators such as Apolipoprotein B [41], Lipoprotein (a) [43], or elevated liver enzymes for confirmation of metabolic dysfunction-associated steatotic liver disease, a condition independently linked with hyperandrogenism (FAI) among women with PMOS [44].

Despite the presentation of greater central adiposity among women with PMOS, cardiometabolic health indicators did not differ between women with and without a diagnosis of PMOS. Given that PMOS is associated with intrinsic insulin resistance [45] and the postmenopausal women with PMOS in this cohort had overweight/obesity and a more central pattern of fat distribution, we expected to see evidence of dyslipidemia and indicators of abnormal glucose regulation. Indeed, this finding was incongruous with previous groups that have reported dyslipidemia or glucose dysregulation among non-obese reproductive-aged women with PMOS [46,47]. Lifestyle behaviors such as diet and physical activity influence cardiometabolic parameters and may be involved [48,49]. However, prescription medication usage may account for these null findings. Women with PMOS reported using a numerically greater, although statistically nonsignificant, number of prescription medications. All participants with PMOS reported currently using at least one prescription medication; in contrast, one-third of the participants without PMOS reported no current prescription medication. When we examined the categories of prescription medications, cardiovascular agents and blood glucose regulators were the two categories most frequently reported by both groups, which further supports the equivocal cardiometabolic health indicators. Cardiovascular agents and blood glucose regulators are among the most common prescription medications reported among US adults over 40 years of age [50] and presumably influenced cardiometabolic health indicators that were measured, including blood lipids, blood pressure, glucose, insulin, and body composition. It is also plausible that antidepressant and anxiolytic medication usage reported by women with and without a PMOS diagnosis also minimized group differences in quality-of-life indicators. The absence of QoL differences should not be interpreted as evidence of equivalence, given the small sample size and potential medication effects.

In this pilot sample, three of nine women with PMOS completed medication reporting using five or more prescription medications. Polypharmacy is a public health concern as it poses a risk for drug interactions, serious side effects, and decreased QoL [51,52,53]. Data gathered from the National Health and Nutrition Examination Survey 2017–2018 report that polypharmacy is present in 20.3% of adult women, 17% of mid-life women (40–64 y), and 45.1% of older women (≥65 y) [54]. The participants with PMOS in this study largely met the definition of midlife (40–64 y) and reported a higher-than-average prevalence of polypharmacy. Future work is needed to confirm the polypharmacy findings from this present study, as well as to consider best practices for health management of postmenopausal women with PMOS. When evaluating and managing cardiometabolic health in postmenopausal women with PMOS, providers should assess whether patients are taking multiple medications as well as consider how these may be combined with lifestyle modifications to improve efficacy.

The cornerstone of PMOS management is lifestyle behavior modification, yet tailored health education for aging women with PMOS is often overlooked. A survey of North American physicians who manage patients with PMOS indicated obesity was the most important long-term concern for this population; yet, inconsistencies in awareness of co-morbidities and recommended management practices, including lifestyle modification, were observed [55]. Importantly, the majority of these providers were gynecologists, reproductive endocrinologists, and infertility specialists who care for women with PMOS during their reproductive years, and the authors noted that it is unclear which practitioner continues to see the aging woman with PMOS [55]. These findings highlight a potential gap in the medical management of women with PMOS as they transition through menopause and signal an opportunity for increased and standardized provider training to bring awareness to health risks for this population. Indeed, Douglas et al. previously reported that postmenopausal women with PMOS exhibit low health literacy and fail to see a PMOS diagnosis as more than a reproductive disorder [56]. To enhance the long-term holistic health outcomes in postmenopausal women with PMOS, clinicians should consider implementing more aggressive screening protocols, earlier preventive interventions, and comprehensive multidisciplinary care approaches.

### Strengths and Limitations

The current study has several strengths, including the recruitment of two groups of postmenopausal women of similar age and BMI category (overweight) with and without PMOS to support the comparison of cardiometabolic health risks. According to BMI, both groups were identified as overweight; anthropometrics and DEXA were performed, and similarly supported evidence of increased visceral body fat deposition, particularly in the PMOS group. This finding was expected and has been correlated with insulin resistance. Evaluation of prescription medication usage and categorization adds valuable insight into the outcomes. To our knowledge, medication usage amongst the postmenopausal women with PMOS has not been reported.

Limitations of this present work include a small sample size and a lack of diversity of the study population. While we purposefully used broad inclusion/exclusion criteria, and recruitment efforts were similar for the entire study sample, we had difficulty recruiting postmenopausal women with a diagnosis of PMOS for a study visit. We were only able to recruit white, non-Hispanic women with a diagnosis of PMOS, resulting in imbalanced groups. An ancillary study of the large, bi-racial cohort recruited from four US cities, the Coronary Artery Risk Development in Young Adults (CARDIA), the CARDIA Women’s Study, similarly reported in imbalanced groups (70% white) when assessing the development of cardiovascular disease among participants who presented with PMOS symptoms decades prior [16]. Additionally, this was a fairly educated sample of women with current healthcare access, so findings may not be generalizable to rural or underserved populations that lack access to healthcare. While all participants completed a screening questionnaire to support the presence or absence of a PMOS diagnosis prior to study start, we acknowledge relying on participant self-report is a major limitation, and the time lapse between diagnosis and pre-menopausal health history data (i.e., Menses and hirsutism) may have influenced participant memory. Modifiable cardiometabolic risk factors, including diet and physical activity, were not assessed. Prescription medication usage was limited to self-report and did not encompass medication usage history or indication. Reliance on self-report of PMOS diagnosis precluded determination of PMOS phenotype, which is associated with varying cardiometabolic risks, and may have contributed to misclassification bias, potentially obscuring true differences.

## 5. Conclusions

These preliminary findings suggest that postmenopausal women with a self-reported history of PMOS may exhibit a more central pattern of fat distribution, despite no significant differences in total fat mass or visceral fat mass, and may report higher prescription medication use than women without a self-reported history of PMOS. Larger, more diverse studies with confirmed PMOS diagnosis, consideration of menopausal stage, and detailed medication data that focus on the use of cardiometabolic medications are needed to determine whether these differences reflect greater cardiometabolic disease burden, treatment patterns, or selection bias.

Future research with larger sample sizes is needed to confirm these preliminary findings and to develop evidence-based guidelines for optimizing long-term metabolic health outcomes in this understudied population. These results underscore the importance of recognizing PMOS as a lifelong metabolic condition that extends beyond reproductive years and requires continued clinical attention throughout the lifespan.

## Figures and Tables

**Table 1:** Participant demographics and health history.

	CON (n = 19) ^a^	PMOS (n = 10) ^b^
Age (y)	56.5 ± 3.6	58.0 ± 5.1
Race (n, %)	16, 84.2% White 1, 5.2% Asian 1, 5.2% Black 1, 5.2% More than one race	10, 100% White
Ethnicity; n, %	15, 78.9% Not Hispanic or Latino 4, 21.1% Hispanic or Latino	9, 88.9% Not Hispanic or Latino 1, 11.1% Hispanic or Latino
Employment Status; n, %	19, 100% Employed for wages	7, 70% Employed for wages 2, 20% Retired 1, 10% Unable to work
Age of menses, y	13.1 ± 2.32	12.9 ± 2.43
Age of PCOS (now PMOS) diagnoses, y	-	23.9 ± 6.64
Age of menopause, y	50.0 ± 4.85	48.4 ± 8.81
Years post-menopause, y	6.5 ± 5.5	9.6 ± 7.9
sFG score ^c^	7.5 ± 2.80	15.4 ± 5.12

^a^ Postmenopausal control. ^b^ Postmenopausal with polyendocrine metabolic ovarian syndrome. ^c^ Simplified Ferriman–Gallwey.

**Table 2:** Participant anthropometrics, sex hormones, cardiometabolic health indicators, and body composition.

	CON ^a^	PMOS ^b^	*p*-Value	Cohen’s d (Effect Size)	95% Confidence Interval
*Anthropometrics*					
Height, m	1.63 ± 0.073	1.62 ± 0.054	0.44		
Weight, kg	72.5 ± 19.3	76.3 ± 12.6	0.52	0.22	[−0.54, 0.98]
BMI, kg/m^2^	25.8 ± 5.3	29.0 ± 3.1	**0.045**	**0.70**	[−0.845, 1.49]
Waist Circumference (in)	35.1 ± 6.88	37.2 ± 4.21	0.31	0.35	[−0.419, 1.22]
Hip Circumference (in)	42.9 ± 5.20	43.1 ± 2.86	0.91	0.039	[−0.72, 0.80]
Waist-to-Hip Ratio	0.81 ± 0.083	0.86 ± 0.065	0.089	**0.64**	[−0.14, 1.42]
*Sex Hormones*					
Estradiol, pg/mL	15.4 ± 1.42	14.99 ± 0	0.21	−0.35	[−1.15, 0.44]
Total Testosterone, ng/mL	20.7 ± 13.5	20.4 ± 14.2	0.94	0.02	[−0.79, −0.74]
Androstenedione, ng/mL	0.40 ± 0.17	0.49 ± 0.17	0.21	0.52	[−0.27, 1.33]
Sex Hormone-Binding Globulin, nmol/L	64.4 ± 32.5	64.4 ± 32.5	0.80	0.10	[−0.66, 0.87]
FAI ^c^	1.40 ± 1.12	1.02 ± 0.59	0.24	−0.38	[−1.16, 0.38]
*Cardiometabolic Health Indicators*					
Systolic Blood Pressure, mmHg	124 ± 15.7	120 ± 10.8	0.53	0.24	[−0.525, 1.01]
Diastolic Blood Pressure, mmHg	69.9 ± 8.6	64.5 ± 8.83	0.12	−0.62	[−1.40, 0.16]
Total Cholesterol, mg/dL	212 ± 31.5	184 ± 40.2	**0.047**	−0.81	[−1.61, 0.02]
VLDL ^d^, mg/dL	23.6 ± 12.8	19.3 ± 7.64	0.34	−0.37	[−1.15, 0.39]
LDL ^e^, mg/dL	115 ± 31.0	103 ± 36.2	0.38	−0.34	[−1.12, 0.42]
HDL ^f^, mg/dL	74.0 ± 25.3	61.0 ± 13.1	0.15	−0.56	[−1.34, 0.21]
Triglycerides, mg/dL	118 ± 63.6	97.3 ± 19.7	0.34	−0.37	[−1.14, 0.39]
Glucose, mg/dL	96.1± 12.6	95.8 ± 19.7	0.95	−0.02	[−0.79, 0.74]
Insulin, µIU/mL	10.1 ± 14.1	9.94 ± 4.36	0.97	−0.01	[−0.81, 0.79]
HbA1c ^g^, %	5.57 ± 0.50	5.76 ± 1.01	0.58	−0.26	[−0.49, 1.03]
hsCRP ^h,^*, mg/L	0.43 ± 0.50	0.60 ± 0.87	0.54	−0.26	[−0.55, 1.07]
*Body Composition*					
Total Fat Mass, g	30.4 ± 12.8	31.1 ± 5.56	0.68	0.06	[−0.70, 0.83]
Total Lean Mass, kg	39.5 ± 6.77	39.7 ± 6.9	0.84	0.022	[−0.74, 0.79]
Percent Fat Mass, %	42.0 ± 7.42	44.0 ± 2.81	0.31	0.31	[−0.46, 1.08]
Trunk Fat Mass, kg	16.6 ± 8.5	17.7 ± 4.02	0.67	0.13	[−0.90, 0.62]
Appendicular Fat Mass, kg	12.9 ± 5.38	12.6 ± 1.83	0.80	−0.76	[−0.84, 0.69]
Visceral Fat Mass, kg	1.07 ± 0.92	1.19 ± 0.35	0.61	0.15	[−0.61, 0.93]
Trunk Fat: Leg Fat Mass	1.61 ± 0.60	1.98 ± 0.40	**0.041**	**0.67**	[−0.12, 1.46]
Trunk Fat: Appendicular Fat Mass	1.20 ± 0.39	1.40 ± 0.21	**0.043**	**0.61**	[−0.18, 1.39]

^a^ Postmenopausal control. ^b^ Postmenopausal with polyendocrine metabolic ovarian syndrome. ^c^ Free Androgen Index. ^d^ Very low-density lipoprotein. ^e^ Low-density lipoprotein. ^f^ High-density lipoprotein. ^g^ Glycosylated hemoglobin. ^h^ High-sensitivity C-reactive protein. * Missing hsCRP data for 5 CON participants due to lab error (hsCRP not measured).

## Data Availability

Deidentified data will be available upon a reasonable request.

## Supplementary Materials

The following supporting information can be downloaded at https://www.mdpi.com/article/10.3390/jcm15145533/s1: Table S1: Quality-of-life and mood questionnaires. Data are reported as T-scores.
