# Supplementary material for: A Pilot Study to Describe Cardiometabolic Health Indicators and Prescription Medication Use in Postmenopausal Women with a Self-Reported History of PMOS
Source: J Clin Med. 2026 Jul 15;15(14):5533. doi: 10.3390/jcm15145533 (PMC13412456; doi:10.3390/jcm15145533)
Supplement: Supplementary file 1 [file jcm-15-05533-s001.zip › jcm-4317566-supplementary.pdf]

Supplemental Table S1. Quality of life and mood questionnaires. Data are reported as T-scores with standard deviation. Group differences were determined with a two-tailed t-test.

	**CON[a] (n=19)**	**PMOS[b] (n=10)**	**p-value**
PROMIS Depression	45.2 ± 4.0	48.2 ± 3.0	0.28
PROMIS Anxiety	50.7 ± 2.5	52.2 ± 2.3	0.54
PROMIS Ability to Participate	53.2 ± 3.1	50.0 ± 2.5	0.32
PROMIS Social Satisfaction	53.5 ± 2.8	54.3 ± 3.0	0.60
PROMIS Sleep Related Impairment	48.1 ± 4.0	51.1 ± 3.6	0.41
PROMIS Sleep Disturbance	49.8 ± 3.5	51.2 ± 3.6	0.69
PROMIS Fatigue	49.8 ± 2.9	51.5 ± 2.5	0.56

[a]Postmenopausal control
[b]Postmenopausal with polyendocrine metabolic ovarian syndrome
